# Seed dormancy and germination vary within and among species of milkweeds

**DOI:** 10.1093/aobpla/ply018

**Published:** 2018-03-02

**Authors:** Thomas N Kaye, Isaac J Sandlin, Matt A Bahm

**Affiliations:** Institute for Applied Ecology, Corvallis, OR, USA

**Keywords:** Cold stratification, conservation biology, habitat restoration, monarch butterfly, plant propagation, pollinator conservation

## Abstract

Pollinators in general and monarch butterflies in particular are in decline due to habitat loss. Efforts to restore habitats for insects that rely on specific plant groups as larvae or adults depend on the ability of practitioners to grow and produce these plants. Monarch larvae feed exclusively on milkweed species, primarily in the genus *Asclepias*, making propagation and restoration of these plants crucial for habitat restoration. Seed germination protocols for milkweeds are not well established, in part due to the large number of milkweed species and conflicting reports of seed dormancy in the genus. We tested for seed dormancy and the optimum period of cold stratification in 15 populations of *A. speciosa* and 1–2 populations of five additional species, including *A. asperula*, *A. fascicularis*, *A. subulata*, *A. subverticillata* and *A. syriaca*. We exposed seeds to cold (5 °C) moist conditions for 0, 2, 4, 6 and 8 weeks and then moved them to 15 °C/25 °C alternating temperatures. In *A. speciosa*, dormancy was detected in eight populations, and this dormancy was broken by 2–4 weeks of cold stratification. The remaining seven populations showed no dormancy. Seed dormancy was also detected in two populations of *A. fascicularis* (broken by 4–6 weeks of cold stratification) and a single population of *A. syriaca* (broken by 2 weeks of cold stratification). No dormancy was detected in *A. asperula*, *A. subulata* or *A. subverticillata*. Seed dormancy appears to be widespread in the genus (confirmed in 15 species) but can vary between populations even within the same species. Variation in seed dormancy and cold stratification requirements within and among *Asclepias* species suggests local adaptation and maternal environments may drive seedling ecology, and that growers should watch for low germination and use cold stratification as needed to maximize seed germination and retain genetic variability in restored populations.

## Teaser

Declines in pollinator populations and monarch butterflies require restoration of habitats with the plants and floral resources that support them. Monarch butterflies in particular lay their eggs exclusively on milkweed species, which their caterpillars require as a food source. Restoring populations of milkweeds can involve planting plugs grown from seed, but recommendations for breaking dormancy and germinating milkweed seeds often disagree. We tested the hypothesis that seed germination and dormancy vary within and among species of milkweed, and found substantial differences across species, and even between populations of the same milkweeds.

## Introduction

Declines in insect pollinator abundance and diversity have been documented over the past decade on regional and global scales (Ghazoul 2005; Steffan-Dewenter *et al.* 2005; Biesmeijer *et al.* 2006; Williams and Osborne 2009; Cameron *et al.* 2011). The loss of these pollinators may have serious impacts on ecologic function and economic stability (Potts *et al.* 2010) because pollinators are important or essential for 65 % of wild flowering plants (Ollerton *et al.* 2011) and 75 % of domestic food crops (Klein *et al.* 2007). Monarch butterflies (*Danaus plexippus*) in particular have declined up to 90 % over the past 15 years (Jepson *et al.* 2015), due in part to loss of milkweed in their summer breeding habitat from increased use of herbicides in intensive farming practices (Brower *et al.* 2012).

Plants in the milkweed family (Asclepiadaceae) are the exclusive food source for monarch butterfly larvae. Monarchs in North America feed on at least 27 species in the genus *Asclepias* and a few closely related genera (Malcolm and Brower 1986). Milkweed populations have suffered considerable declines in habitat in the central USA due mainly to the use of genetically modified, glyphosate resistant crops, and the placement of an additional 10 million hectares of herbicide-tolerant corn into production since 2007 (Pleasants and Oberhauser 2012). Restoring milkweed habitat is crucial to the recovery of the monarch butterfly and will require planting large numbers of milkweeds throughout its geographic range (Fallon *et al.* 2015; Jepsen *et al.* 2015; Luna and Dumroese 2013). Milkweeds also benefit many other insects and pollinators, such as milkweed beetles in the Cerambycidae (Evans 2014), bumblebees (*Bombus* spp.), honey bees (*Apis mellifera*), and other bees and lepidoptera of various body sizes (e.g. Fishbein and Venable 1996), so conservation of these plants has cascading positive effects on ecosystem function and multiple groups of organisms (Buckley and Nabhan 2016; Dumroese *et al.* 2016).

To produce milkweeds for habitat conservation at a large scale, reliable propagation protocols must be available. One method of milkweed propagation is by vegetative rhizome cutting, which can be an effective method for growing and establishing several species of milkweeds in gardens and habitat restoration sites (Ecker and Barzilay 1993; Landis 2014; Landis and Dumroese 2014). Although cuttings of this type can produce large plants relatively quickly, the process generally results in clones of fewer genotypes, and thus lower genetic diversity and fitness in restored populations, than propagation from seed (Williams 2001). Germination is the first step in producing plants from seed and requires an understanding of dormancy and germination requirements for efficient and successful plant propagation at a large scale. But seed germination protocols for *Asclepias* species are relatively poorly developed, despite the large number of species in the genus. Over 100 species of *Asclepias* are known to occur in North and Central America (Mabberley 1997) with ~76 species in the USA and Canada (USDA, NRCS 2017). Related species, and populations within species, are often assumed to have the same germination requirements because of phylogenetic constraints (Seglias *et al.* 2018). It is generally unknown if seed dormancy and cold stratification requirements vary across *Asclepias* species or populations. In some cases, suggested germination protocols for *Asclepias* species are inconsistent or even contradictory (Borland 1987). For example, cold stratification has been recommended to release seeds of *A. speciosa* from dormancy but some practitioners report that no cold stratification is necessary for large-scale propagation (Stevens 2000).

Here we present the results of research on seed dormancy and germination in six *Asclepias* species. In particular, we examine variation in dormancy and cold stratification requirements among populations of *A. speciosa* as well as between five other species. We test the hypothesis that dormancy varies among populations of *A. speciosa* from across the species’ geographic range, and populations of the five additional species. We predict that dormancy varies substantially, thus explaining conflicting reports of the importance of cold stratification in this genus. We also compile and review the primary literature and unpublished reports on dormancy and germination of several additional milkweed species.

## Methods

### Germination and viability tests

**Figure 1.**
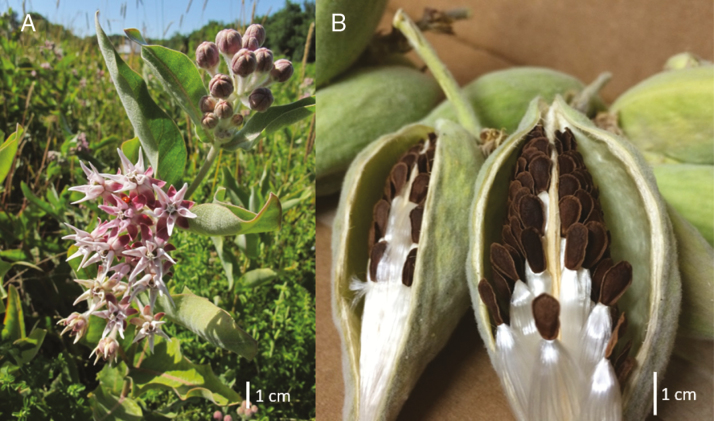
*Asclepias speciosa* (A) in flower and (B) dehiscing fruit with seeds. Plant photographed in Willamette Valley, OR.

**Figure 2.**
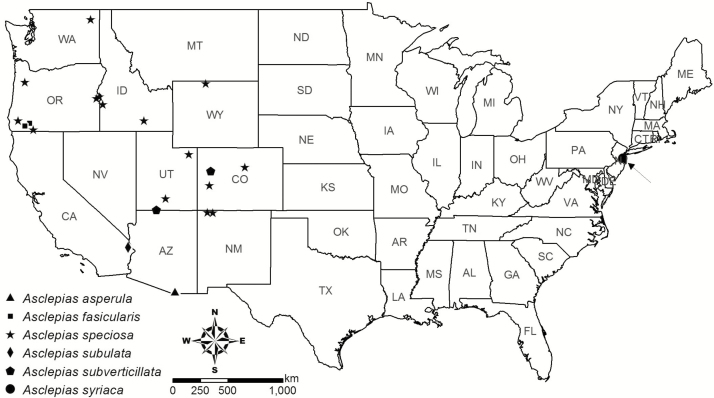
Location in the USA of each *Asclepias* source population included in this study. The arrow indicates a population of *A. syriaca* from New Jersey.

We obtained seed from 15 populations of *A. speciosa* (Fig. 1) and one or two populations of five additional North American species, including *A. asperula*, *A. fascicularis*, *A. subulata*, *A. subverticillata* and *A. syriaca* (Table 1) from multiple locations in the USA (Fig. 2). These seeds had either been collected during the summer immediately prior to germination testing, or had been in dry, cold (<0 °C) storage at the US Forest Service Bend Seed Extractory or the US Agricultural Research Service, National Plant Germplasm System.

**Table 1.** *Asclepias* species and populations included in dormancy and germination tests, with seed sources, samples (50 seeds each) per treatment and years in storage. ^†^BSE indicates the US Forest Service, Bend Seed Extractory; GRIN indicates the US National Germplasm Resources Information Network.

Species	Population	Seed source^†^ (accession number)	Samples per treatment	Years in storage
*Asclepias speciosa*	Denver, CO	BSE (CO932-306-Jefferson-12)	4	5
	Montrose, CO	Colorado Plateau Native Plant Program	4	2
	Minidoka, ID	US Fish and Wildlife Service	5	0
	Treasure Valley, ID	Idaho State University	5	0
	Navajo Dam, NM	BSE (NM930N-86-SanJuan-12)	4	5
	Vernal, NM	BSE (SOS-NM930N-12-10)	4	7
	Galice, OR	BSE (SOS-OR110-904-Josephine-15)	4	2
	Malheur, OR	US Fish and Wildlife Service	5	0
	Ontario, OR	Institute for Applied Ecology	5	0
	Tub Springs, OR	BSE (OR110-637-Jackson-13)	4	4
	Willamette Valley, OR	Heritage Seedlings, Inc.	5	0
	Escalante, UT	BSE (UT030-256-Garfield-15)	4	2
	Maeser, UT	BSE (SOS-UT080-140-UINTAH-13)	4	4
	Little Pend Oreille, WA	US Fish and Wildlife Service	5	0
	Kane, WY	BSE (SOS-WY020-11-11)	4	10
*Asclepias asperula*	Santa Cruz, AZ	GRIN (W6-48232)	4	4
*Asclepias fascicularis*	Agate Reservoir, OR (1)	BSE (SOS-OR110-608-Jackson-13)	4	4
	Gold Hill, OR (2)	BSE (SOS-OR110-968-Jackson-15)	4	2
*Asclepias subulata*	Lake Havasu City, CA	GRIN (W6-46777)	4	5
*Asclepias subverticillata*	Anvil Points, CO	BSE (SOS-CO932-160-08)	4	9
	UT	GRIN (W6-36792)	4	10
*Asclepias syriaca*	NJ	GRIN (W6-48817)	4	4

To determine if seeds from each species and source population required a period of cold stratification to release them from dormancy, we exposed seeds to a range of cold, moist periods. Dormancy was defined here as the inability of a seed to germinate in a specified period of time under combinations of normal physical factors (e.g. temperature, light, etc.) that are otherwise favourable for germination, following Baskin and Baskin (2004). Seeds were stratified for 0, 2, 4, 6 or 8 weeks at 5 °C at the Oregon State University Seed Lab. We used four or five replicates (depending on seed availability, Table 1) of 50 seeds from each seed source. For all replicates, seeds were placed on moistened germination paper in 15 cm × 15 cm × 3 cm transparent plastic boxes with fitted lids. The paper was moistened weekly as needed with distilled water. After stratification treatments were applied, seeds were placed in a growth chamber with 15 °C/25 °C alternating temperatures, with 8 h of darkness at 15 °C and 16 h of fluorescent light at 25 °C. We defined germination as emergence of the radicle at least 3 mm, and counted germinated seeds after 10 days (Baskin and Baskin 2001). In preliminary trials (not shown) we found that 95 % or more seeds germinated within this period of time, and any additional germination took many weeks to complete. Seed viability was tested with tetrazolium (TZ) by the Oregon State University Seed Lab using standard procedures (Elias *et al.* 2012). The TZ test was used to estimate the percentage of live and dead seeds in each seed source, regardless of dormancy level (Baskin and Baskin 2001). Seeds were cut longitudinally to expose interior tissues and facilitate the entrance of TZ solution, and incubated in a 1 % TZ solution for 24 h at 35 °C. Seeds were inspected for TZ staining, specifically the pattern and intensity of red colour in live tissues in seeds. Seed with uniform stain colour were recorded as viable seeds. Tetrazolium tests were performed with samples of 157 to 210 seeds per source population.

### Analysis

We tested for effects of cold stratification and population with a general linear model of analysis of variance, with cold stratification treatment as a fixed effect and seed source as a random effect using NCSS statistical software (Hintze 2008). Separate analyses were performed for *Asclepias speciosa* populations, and the five additional *Asclepias* species and their populations. Data are reported with the mean ± 95 % confidence interval. Confidence intervals for the viability estimates were calculated from the normal approximation of the binomial distribution using the proportion of seeds that were considered viable, the proportion of seeds that were considered non-viable and the total number of seeds tested.

## Results

### Intraspecific population differences in dormancy and germination of *A. speciosa*

**Figure 3.**
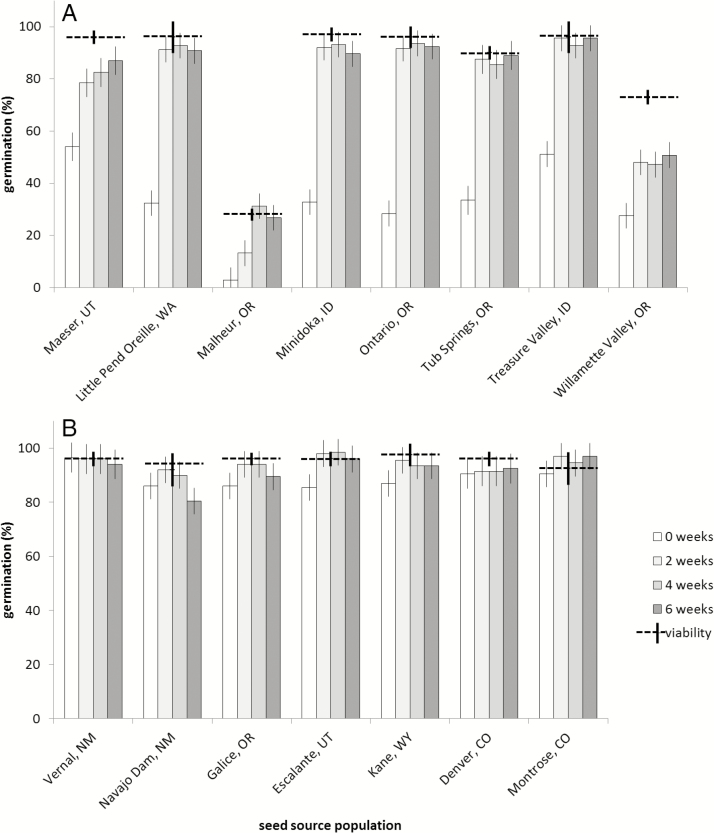
Seed germination in response to different periods of cold stratification for 15 populations of *Asclepias speciosa* (A) dormant seeds and (B) non-dormant seeds. Seed viability as estimated with TZ is shown with horizontal hatched lines. Error bars represent 95 % confidence intervals.

There was a significant stratification treatment by population interaction (*F* = 17.62, df = 56;255, *P* < 0.0001) for *A. speciosa*, with substantial improvement in germination after stratification for eight out of the 15 populations tested (grouped for ease of visual inspection in Fig. 3A), but only slight or no dormancy in the remaining seven populations (Fig. 3B). Seed viability was over 90 % in most *A. speciosa* populations as tested with TZ, with the exception of two populations with lower viability, Willamette Valley, OR (73 ± 6.0 %) and Malheur, OR (28 ± 6.1 %). In populations with substantial dormancy, <60 % of seeds germinated without cold stratification, but with 2 or more weeks of cold stratification germination increased to the levels of seed viability, or nearly so. For example, without cold stratification 32 ± 5 % of seeds from Little Pend Oreille, WA, germinated, but after 2 weeks of cold stratification germination rose to 91 ± 5 %, close to the viability estimate of 96 ± 2.6 % (Fig. 3A). Seed from Malheur, OR, required 4 weeks of cold stratification to fully break dormancy, while germination of seeds from Willamette Valley, OR, did not germinated to the level of their estimated viability. Populations with largely non-dormant seeds (Fig. 3B) germinated to very high rates at or near their viability estimate without any cold stratification. Seed germination was not higher after 8 weeks of cold stratification than 6 weeks in any population tested, so this treatment is not shown (Fig. 3).

### Interspecific differences in dormancy and germination

**Figure 4.**
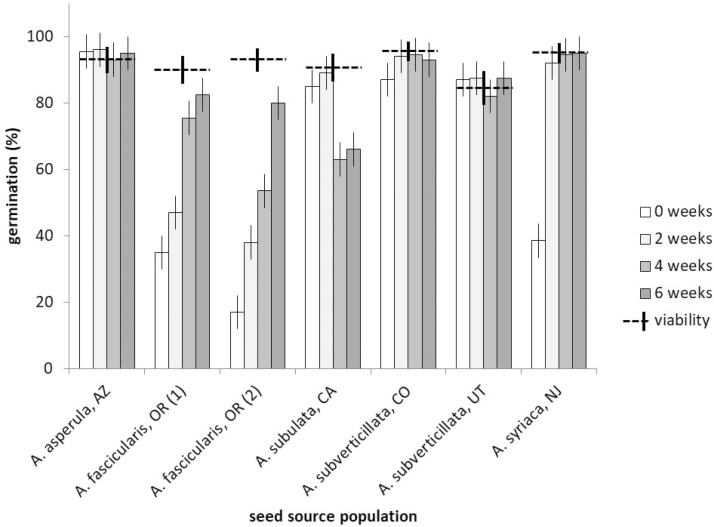
Seed germination in response to difference periods of cold stratification for five *Asclepias* species from the USA. Seed viability as estimated with TZ is shown with horizontal hatched lines. Error bars represent 95 % confidence intervals.

Dormancy and germination varied among species of *Asclepias* examined here. Again there was a significant interaction between cold stratification treatment and population for the five taxa examined (*F* = 40.29, df = 24;105, *P* < 0.00001), with two species showing positive effects on germination of cold stratification of 2 or more weeks, but the remaining taxa possessing little or no dormancy. Without cold stratification, both populations of *Asclepias fascicularis* that were sampled had germination of only 17 ± 5.1 % and 35 ± 5.1 %, despite viability estimates of 93 ± 3.5 % and 90 ± 4.2 %, respectively, indicating substantial dormancy. Both of these populations required 6 weeks of cold stratification to achieve maximum germination of 80 ± 5.1 % and 82.5 ± 5.1 %, respectively (Fig. 4). Eight weeks of cold stratification (not shown) did not improve seed germination any further in *A. fascicularis*. *Asclepias syriaca* also had partial dormancy, with only 38.5 % of seeds germinating without cold stratification, despite viability estimated at 95 ± 3.0 %. After 2 weeks of cold stratification of this sample, germination increased to 92 ± 5.1 % (Fig. 4). The remaining species, *A. asperula*, *A. subulata* and *A. subverticiallata*, germinated to levels close to (or within 95 % confidence intervals) of their seed viability as estimated by TZ even without cold stratification. Again, cold stratification for 8 weeks (not shown) did not increase seed germination over the 6-week period (Fig. 4).

## Discussion

### Seed dormancy and viability

We found that seed dormancy varied widely within and among *Asclepias* species, and that the period of time in cold stratification needed to break dormancy varied as well. In *A. speciosa* in particular, seeds from seven source populations had essentially no dormancy, while seeds from eight other populations generally needed 2 weeks (but up to 6 weeks) of cold stratification to reach germination levels similar to viability. Of the remaining species tested here, the seeds of three (*A. asperula*, *A. subulata* and *A. subverticillata*) were non-dormant, while two (*A. fascicularis* and *A. syriaca*) required cold stratification to break dormancy. Seed germination tests in *A. subulata* have also found no dormancy in populations from California (Everett 2012). Previous reports have suggested little or no dormancy in *A. speciosa* (Bartow 2006; Skinner 2008), but our results indicate that the benefits of cold stratification differ substantially among source populations. Even in populations where seed germination was enhanced by cold stratification, many seeds germinated without a cold treatment, suggesting that when dormancy was present, it typically affected only a portion of the seeds from a given location. This was true when dormancy was present in *A. speciosa* as well as *A. fascicularis* and *A. syriaca*, which had 3 % to 54 % germination even without cold stratification.

Seed viability was typically very high (over 85 %) in our samples, with two exceptions out of the 22 populations we tested. High seed viability has also been reported in *A. cordifolia* (Barner 2009) and *A. syriaca* (Bhowmik 1978). Unpublished records of seed viability and dormancy in *Asclepias* species tested by the US Department of Agriculture National Laboratory for Genetic Resources Preservation are compiled in Table 2. Their records from germination tests of small seed samples (typically two replicates of 50 seeds, but in one case only 44 seeds) with specific periods of cold stratification ranging from 0 to 21 days suggest wide variation in viability and dormancy within and among populations of nine *Asclepias* species. For example, among eight populations of *A. fascicularis*, seed viability ranged from 68 to 98 %, and seven of the source populations possessed no seed dormancy (defined here as <80 % germination of viable seeds) when no cold stratification was provided. This is in contrast to our findings of substantial dormancy in both populations of *A. fascicularis* that we examined. In *A. speciosa*, seed viability ranged from 65 to 98 % among 13 populations, and six out of nine populations were dormant without cold stratification (Table 2), a pattern that agrees with our observation of wide variation in dormancy in that species.

**Table 2.** Summary of seed dormancy in *Asclepias* species tested at the USDA/ARS National Laboratory for Genetic Resources Preservation, Seed Quality Lab, with duration of cold stratification (if any), number of populations sampled and number of populations with and without dormancy. Germination is relative to viability, and is shown as the amount or range determined for each period of stratification. Populations were classified as possessing seed dormancy if <80 % of viable seeds germinated. Each population represents a separate seed collection.

Species	Stratification	No. of populations sampled	Viability	Populations without dormancy		Populations with dormancy	
				No. of populations	Germination	No. of populations	Germination
*A. asperula*	7 days	1	100 %	1	100 %	0	–
*A. fascicularis*	0 day	8	68–98 %	7	92–100 %	1	37 %
*A. hirtella*	0 day	1	100 %	0	–	1	33 %
*A. latifolia*	0 day	1	100 %	1	100 %	0	–
*A. speciosa*	0 day	9	73–98 %	3	89–100 %	6	8–78 %
	7 days	4	65–95 %	4	93–100 %	0	–
	14 days	1	98 %	1	100 %	0	–
*A. subulata*	0 day	1	100 %	1	100 %	0	–
*A. subverticillata*	0 day	2	92–96 %	2	100 %	0	–
	14 days	1	96 %	1	100 %	0	–
*A. syriaca*	0 day	8	92–100 %	0	–	8	10–67 %
*A. tuberosa*	0 day	3	96–100 %	1	88 %	2	50–72 %
	7 days	1	55 %	1	93 %	0	–
	14 days	1	98 %	0	–	1	50 %
	21 days	3	50–76 %	3	100 %	0	–

### Cold stratification

Baskin and Baskin (2001) suggest that *Asclepias* seeds may have physiological dormancy, which is typically broken by cold stratification, seed coat removal or chemical inducements, all of which improve germination in *A. syriaca* (Oegema and Fletcher 1972). Our review of published and unpublished accounts of seed germination and dormancy in 17 species of *Asclepias* suggests that variation in the period of cold stratification needed to break dormancy appears to be common within and among milkweed species (Table 3). For example, Green and Curtis (1950) examined five species of milkweed from Wisconsin and found a range of periods of cold stratification needed to completely release seeds from dormancy, from none at all to 5 months. Most researchers use cold stratification temperatures of 4 °C to 5 °C (Luna and Dumroese 2013), although some placed seeds outdoors in winter to expose seeds to ambient cold conditions (e.g. Green and Curtis 1950).

**Table 3.** Duration of cold stratification needed to break dormancy in *Asclepias* species from published sources, with post stratification temperatures used or recommended for germination, where reported.

Species	Period of cold stratification needed to break dormancy	Germination temperature	References
*Asclepias amplexicaulis*	60 days		Heon and Larsen (1999)
*Asclepias erosa*	0 day		Everett (2012)
*Asclepias exaltata*	0–60 days		Heon and Larsen (1999)
*Asclepias floridiana*	120 days	18–21 °C	Green and Curtis (1950)
*Asclepias incarnata*	0–60 days		Heon and Larsen (1999)
	5 days	27 °C/16 °C	Lincoln (1983)
	7 days	18 °C	Schultz *et al.* (2001a)
	30 days		Vandevender and Lester (2014a)
	90 days		Cullina (2000)
*Asclepias hirtella*	0–60 days		Heon and Larsen (1999)
*Asclepias meadii*	0 day		Bowles *et al.* (1998)
	70 days		Betz (1989)
*Asclepias ovalifolia*	120 days		Green and Curtis (1950)
*Asclepias perennis*	0 day	10–30 °C	Edwards *et al.* (1994)
*Asclepias purpurascens*	0–60 days		Heon and Larsen (1999)
*Asclepias speciosa*	0 day	21 °C/10 °C	Bartow (2006)
	0 day		Skinner (2008)
*Asclepias subulata*	0 day		Everett (2012)
*Asclepias sullivantii*	0–60 days		Heon and Larsen (1999)
	90–120 days	21–27 °C/18–24 °C	Blessman *et al.* (2002)
	150 days		Green and Curtis (1950)
*Asclepias syriaca*	0 day	26 °C/21 °C	Radivojevic *et al.* (2016)
	0–60 days		Heon and Larsen (1999)
	7 days	20 °C/30 °C	Evetts and Burnside (1972)
	7 days	20 °C/30 °C	Farmer *et al.* (1986)
	14–21 days	30 °C/15 °C	Baskin and Baskin (1977)
	21 days	25 °C	Jeffery and Robison (1971)
	30 days	18 °C	Schultz *et al.* (2001b)
	30 days	20 °C/30 °C	Lincoln (1976)
	56 days	26 °C	Oegema and Fletcher (1972)
	60 days		Green and Curtis (1950)
*Asclepias tuberosa*	0 day		Phillips (1985)
	0 day		Green and Curtis (1950)
	0–60 days		Heon and Larsen (1999)
	21 days	30 °C/10 °C	AOSA (2016)
	30 days		Vandevender and Lester (2014b)
	60 days		Bir (1986)
	60–90 days		Grabowski (1996)
	71 days		Nichols (1934)
	90–120 days	21–27 °C/18– 24 °C	Blessman and Flood (2001)
	105 days	33 °C/19 °C or 26 °C	Salac and Hesse (1975)
*Asclepias verticillata*	0–60 days		Heon and Larsen (1999)
	30 days		Green and Curtis (1950)
*Asclepias viridiflora*	0–60 days		Heon and Larsen (1999)

Species with non-dormant seeds (i.e. in which no benefit of cold stratification has been reported) include *A. erosa* (Everett 2012), *A. meadii* (Bowles *et al.* 1998), *A. perennis* (Edwards *et al.* 1994), *A. speciosa* (Bartow 2006; Skinner 2008), *A. syriaca* (Radivojevic *et al.* 2016) and *A. tuberosa* (Green and Curtis 1950; Phillips 1985). However, in some of these same species cold stratification has been found to improve germination in studies from other populations, and the optimal period of cold stratification varies as well. For example, *A. syriaca* and *A. tuberosa* are important for monarch butterfly populations (Seiber *et al.* 1986; Borders and Lee-Mader 2014) and have received the most attention for their germination requirements, and both vary widely in cold stratification needs. *Asclepias syriaca* populations with seed dormancy may require 56 days or more of cold stratification (Green and Curtis 1950; Oegema and Fletcher 1972), or as little as 7 days (Evetts and Burnside 1972; Farmer *et al.* 1986). We found 2 weeks of cold stratification was sufficient to release dormancy in this species, but we did not try a shorter period. In *A. tuberosa*, as much as 90–120 days of cold stratification (Salac and Hesse 1975; Cullina 2000; Blessman and Flood 2001) or as little as 21 days (AOSA 2016) may be needed for optimal germination of dormant seed lots. Similarly, 30 days (Vandevender and Lester 2014a) down to 1 week or less (Lincoln 1983; Schultz *et al.* 2001a) of cold stratification may be needed to improve germination in *A. incarnata*. Among species tested by the National Laboratory for Genetic Resources Preservation, most populations that received cold stratification of 7–21 days were released from dormancy (Table 2).

### Factors that affect dormancy and germination in *Asclepias*

Seed dormancy can vary among species, populations (Keith and Myerscough 2016; Siles *et al.* 2017), collections from the same population but different years (Green and Curtis 1950; Kaye 1999) and among individual mother plants (Andersson and Milberg 1998). Seed dormancy can affect interactions within and among species by determining the seasonal timing of germination, seedbank dynamics, and exposure of seeds and seedlings to hazards and competition for resources (Harper 1977; Baskin and Baskin 2001). Dormancy has been shown to be under genetic control in some species, often in response to natural selection, such as in *Digitaria* (Hacker 1984), *Arabidopsis* (Alonso-Blanco *et al.* 2003; Bentsink *et al.* 2010) and *Oryza* (Gu *et al.* 2004), or it can result from conditions during seed maturation in the environment of the maternal plant and zygote (Bodrone *et al.* 2017; Penfield and MacGregor 2017), or both (Postma and Agren 2015). To our knowledge, neither genetic nor environmental controls on dormancy have been documented in *Asclepias*. Despite the widespread presence of seed dormancy in *Asclepias* species, persistent seed banks have not been detected even when milkweeds are present in the above-ground vegetation (Smith and Kadlec 1983; Johnson and Anderson 1986). Seedling emergence of *A. syriaca* exceeds 80 % for seeds buried 0.5–4 cm (Yenish *et al.* 1996) leaving only a small proportion of seeds in the soil unaccounted for and which could contribute to a seedbank or succumb to mortality. Germination of *Asclepias* seeds can also be affected by light, scarification, substrate, temperatures, after-ripening and other factors. Light may be required for germination in some *Asclepias* species, such as *A. incarnata* (Lincoln 1976; Deno 1993), or have no effect on others, including *A. tuberosa* (Mitchell 1926; Deno 1993). Light has little or no effect on germination of *A. syriaca* once adequate (≥2 weeks) cold stratification has been provided (Baskin and Baskin 1977; Lincoln 1983). Mechanical scarification of the seed coat improves germination in *A. syriaca* (Oegema and Fletcher 1972; Evetts and Burnside 1972). Smoke treatments that mimic wildfire smoke can improve germination of *A. syriaca* as well (Mojzes and Kalapos 2015), but burning and soil disturbance reduced seedling emergence in *A. meadii* (Roels 2013). Experiments with temperature and substrate have found that *A. syriaca* germinates best under alternating temperatures of 20 °C/30 °C on clay or clay mixed with peat (Farmer *et al.* 1986), or 21 °C/26 °C on sand or loam (Radivojevic *et al.* 2016). Temperatures that promote successful germination after cold stratification of *Asclepias* also vary considerably, and most reports recommend an alternating temperature regime. For example, a temperature cycle of 10 °C/30 °C is effective for *A. perennis* (Edwards *et al.* 1994) and the standard for seed testing in *A. tuberosa* (AOSA 2016). Alternating 16 °C/27 °C (Lincoln 1983) has been successfully used for *A. incarnata*, and cycles of 15 °C/30 °C (Baskin and Baskin 1977) and 20 °C/30 °C (Evetts and Burnside 1972; Lincoln 1976; Farmer *et al.* 1986) have been recommended for *A. syriaca*. Bhowmik found germination in *A. syriaca* increased with increasing temperature from 10 °C to 27 °C. We used alternating 15 °C/25 °C in our trials and this cycle was generally effective for germination of the species and populations we examined. In the case of the *A. speciosa* population from Malheur, OR, we also used a cycle of 20 °C/30 °C but found germination to be reduced by about 30 % compared to the 15 °C/25 °C cycle (data not shown).

After-ripening, a period of time after seed dispersal in which changes in the seeds affect their ability to germinate, can affect some species of *Asclepias*. For example, Baskin and Baskin (1977) suggest that with after-ripening *A. syriaca* seeds can germinate at lower temperatures and after shorter periods of cold stratification, and Bhowmik (1978) found that germination increased gradually with time in storage from 1 to 11 months. Seed dormancy in *A. speciosa* populations included in our experiments may have been affected by the amount of time seeds were in storage as well as local environmental conditions. We used fresh seeds that had been collected in the same year of the experiment and had been stored for ~3 months, as well as seeds that had been in dry, cold (<0 °C) storage for up to 6 years. Fresh seeds from six populations had >40 % dormancy, while seeds stored for 2, 5 and 6 years had <15 % dormancy. But seeds stored for 4 years from two populations had dormancy levels similar to fresh seeds, suggesting that dormancy can persist even in stored seeds and may involve other factors beyond storage. Further confounding the effects of storage time was that most of our fresh seeds came from latitudes north of 40° and all of the stored seeds came from farther south, and no seed dormancy was found in the southern populations; in other words, most non-dormant seeds had been stored and were from southern latitudes. Therefore, it is not possible for us to separate the effects of storage time from local environments, but we speculate that both seed storage and local conditions (Seglias *et al.* 2018) could influence seed dormancy in *A. speciosa* and possibly other *Asclepias* species.

## Conclusions

Growers of *Asclepias* species should consider cold stratification to break seed dormancy when it is encountered. We found widespread evidence of seed dormancy in the genus through germination tests reported here as well as published and unpublished reports. Seed dormancy has been detected in at least some populations of 15 species in the genus, including *A. amplexicaulis*, *A. exaltata*, *A. fascicularis*, *A. floridiana*, *A. incarnata*, *A. hirtella*, *A. meadii*, *A. ovalifolia*, *A. purpurascens*, *A. speciosa*, *A. sullivantii*, *A. syriaca*, *A. tuberosa*, *A. verticillata* and *A. viridiflora* (Table 3). Cold stratification is a relatively simple method to break this dormancy and increase seed germination substantially, and can shorten germination time (Salac and Hesse 1975) and even out differences in germination across populations (Milberg and Andersson 1998). For native plant specialists, commercial growers and managers who want to grow milkweed to support habitat restoration for monarch butterflies (Borders and Lee-Mader 2015) and pollinators, the use of cold stratification treatments may be integral to successful large-scale production. For most milkweed species and populations, some germination of seeds is likely to occur even in the absence of cold stratification, but incomplete germination could result in loss of genetic variability in plants or harvested seeds and reduced adaptive potential of restored populations in the long term (Basey *et al.* 2015). Variation in seed dormancy and cold stratification requirements among *Asclepias* species and populations suggests that growers should watch for low seedling emergence and use cold stratification as needed to maximize seed germination. Seed dormancy in *Asclepias* may be under the control of multiple factors, including local adaptation, maternal response to specific environments, as well as storage conditions, with implications for plant propagation and ecological interactions across this genus of flowering plants.

## Sources of Funding

This work was supported by a US Fish and Wildlife Service Cooperative Agreement to T.N.K. at the Institute for Applied Ecology.

## Contributions by the Authors

The germination experiments were designed by T.N.K. and implemented by T.N.K., I.J.S. and M.A.B. T.N.K. conducted the data analysis. All authors contributed to the literature review and writing.

## Conflict of Interest

None declared.
